# Severe Tuberculosis in Humans Correlates Best with Neutrophil Abundance and Lymphocyte Deficiency and Does Not Correlate with Antigen-Specific CD4 T-Cell Response

**DOI:** 10.3389/fimmu.2017.00963

**Published:** 2017-08-21

**Authors:** Alexander V. Panteleev, Irina Yu Nikitina, Irina A. Burmistrova, George A. Kosmiadi, Tatyana V. Radaeva, Rasul B. Amansahedov, Pavel V. Sadikov, Yana V. Serdyuk, Elena E. Larionova, Tatef R. Bagdasarian, Larisa N. Chernousova, Vitaly V. Ganusov, Irina V. Lyadova

**Affiliations:** ^1^Immunology Department, Central Tuberculosis Research Institute, Moscow, Russia; ^2^Physiatry Department, Central Tuberculosis Research Institute, Moscow, Russia; ^3^Radiology Department, Central Tuberculosis Research Institute, Moscow, Russia; ^4^Microbiology Department, Central Tuberculosis Research Institute, Moscow, Russia; ^5^Department of Microbiology, University of Tennessee, Knoxville, TN, United States

**Keywords:** tuberculosis, Th1 cells, pulmonary destruction, neutrophils, band neutrophils, polyfunctional lymphocytes

## Abstract

It is generally thought that *Mycobacterium tuberculosis* (*Mtb*)-specific CD4^+^ Th1 cells producing IFN-γ are essential for protection against tuberculosis (TB). In some studies, protection has recently been associated with polyfunctional subpopulation of *Mtb*-specific Th1 cells, i.e., with cells able to simultaneously secrete several type 1 cytokines. However, the role for *Mtb*-specific Th1 cells and their polyfunctional subpopulations during established TB disease is not fully defined. Pulmonary TB is characterized by a great variability of disease manifestations. To address the role for *Mtb*-specific Th1 responses during TB, we investigated how Th1 and other immune cells correlated with particular TB manifestations, such as the degree of pulmonary destruction, TB extent, the level of bacteria excretion, clinical disease severity, clinical TB forms, and “Timika X-ray score,” an integrative parameter of pulmonary TB pathology. In comparison with healthy *Mtb*-exposed controls, TB patients (TBP) did not exhibit deficiency in *Mtb*-specific cytokine-producing CD4^+^ cells circulating in the blood and differed by a polyfunctional profile of these cells, which was biased toward the accumulation of bifunctional TNF-α^+^IFN-γ^+^IL-2^−^ lymphocytes. Importantly, however, severity of different TB manifestations was not associated with *Mtb*-specific cytokine-producing cells or their polyfunctional profile. In contrast, several TB manifestations were strongly correlated with leukocyte numbers, the percent or the absolute number of lymphocytes, segmented or band neutrophils. In multiple alternative statistical analyses, band neutrophils appeared as the strongest positive correlate of pulmonary destruction, bacteria excretion, and “Timika X-ray score.” In contrast, clinical TB severity was primarily and inversely correlated with the number of lymphocytes in the blood. The results suggest that: (i) different TB manifestations may be driven by distinct mechanisms; (ii) quantitative parameters and polyfunctional profile of circulating *Mtb*-specific CD4^+^ cells play a minor role in determining TB severity; and (iii) general shifts in production/removal of granulocytic and lymphocytic lineages represent an important factor of TB pathogenesis. Mechanisms leading to these shifts and their specific role during TB are yet to be determined but are likely to involve changes in human hematopoietic system.

## Introduction

Tuberculosis (TB) is currently the deadliest infectious disease of humans (1). In 2015, there were an estimated 10.4 million new TB cases worldwide and 1.8 million TB deaths including 0.4 million deaths resulting from TB among people living with HIV (1). Global TB control critically depends on efficient TB prevention and treatment. Elaboration of new strategies for TB prevention and treatment will likely benefit from deeper understanding of mechanisms that protect host against TB onset and mediate protection or pathology during TB disease.

It is generally assumed that host protection against TB relies on IFN-γ-producing CD4^+^ T cells that are able to activate macrophages for *Mycobacterium tuberculosis* (*Mtb*) killing (2, 3). The concept is supported by increased severity of *Mtb* infection in mice deficient in CD4^+^ cells or in IFN-γ and by increased risk of mycobacterial infections in humans infected with HIV or bearing mutations in IL-12/IFN-γ axis (4–7). However, recent experimental data have brought questions regarding the role for Th1/IFN-γ in TB protection. Particularly, in several experimental models, the levels of Th1/IFN-γ responses measured following vaccination or CD4^+^ T-cell depletion did not correlate with a degree of protection evaluated as *Mtb* load or survival time of *Mtb*-infected animals (8–13). In one study, IFN-γ-mediated protection was attributed to the inhibition of deleterious Th17 response instead of suppression of *Mtb* replication (14). Some examinations showed a deleterious effect of uncontrolled CD4^+^ T-cell reactivity (15, 16). In an elegant study by Sakai et al., IFN-γ accounted for only ~30% of the cumulative CD4^+^ T-cell-mediated reduction in murine lung bacterial loads, and overproduction of IFN-γ by individual CD4^+^ T cells was lethal (16).

The role for Th1/IFN-γ in *Mtb* control in humans is even less clear. In children, the frequency and cytokine profile of *Mtb*-specific CD4^+^ T cells generated in response to BCG vaccination did not correlate with the protection against TB (17). Comparison of Th1/IFN-γ levels in TB patients (TBP) and individuals with latent TB infection (LTBI) did not reveal stable differences between the two groups (18–23). Interferon-gamma-release assays do not discriminate LTBI and active TB reliably (24–26). These data suggest that in most cases, TBP do not exhibit severe or obvious Th1/IFN-γ deficiency. However, whether quantitative parameters of Th1/IFN-γ responses affect TB severity is not fully clear.

Recent analyses of CD4^+^ T-cell functional heterogeneity led to the identification of T-cell populations which differ in their ability to simultaneously secrete IFN-γ, TNF-α, and/or IL-2. Data on the protective efficacy of *Mtb*-specific T cells secreting different combinations of IFN-γ, TNF-α, and IL-2 and the prevalence of these cells during LTBI or active TB are contradictory (27). In particular, some studies reported an association of polyfunctional TNF-α^+^IFN-γ^+^IL-2^+^ lymphocytes with protective immunity to TB and the persistence of latency (28, 29) and found increased proportions of monofunctional TNF-α^+^ or bifunctional TNF-α^+^IFN-γ^+^ CD4^+^ T cells in subjects with active TB (28–31). In other studies, in contrast, polyfunctional cells were more frequent in TBP, while LTBI subjects had significantly higher proportions of mono- and bifunctional populations, including IFN-γ^+^ and IFN-γ^+^IL-2^+^ (32–35). Some authors did not find significant differences between TBP and LTBI subjects in polyfunctional lymphocytes (35). Finally, antigens used for cell stimulation and stimulation duration may have contributed to this controversy (35, 36). Overall, the role for Th1-cell functional heterogeneity in TB defense and immunological correlates of TB protection or pathology remain unclear.

Latent TB infection and sputum-positive (active) TB represent two polar states of *Mtb* infection (37, 38). Comparison of immune responses during LTBI and sputum-positive TB represents one of the most common approaches used to identify the basis of immune protection during *Mtb* infection (18, 19, 39–42). However, active infection affects immune system and, therefore, comparison of immune responses during LTBI and TB may in some cases be inappropriate. Specifically, immunological differences seen between LTBI subjects and TBP may represent a result of different infection activity and do not necessarily mark protective and pathological signatures. Another important point is that there is a broad spectrum of pathology states between LTBI and sputum-positive TB (37). Particularly, active TB has a great variability of clinical manifestations that include diversities in the forms of pulmonary pathology, pulmonary destruction, disease extent, bacteria excretion, and other characteristics (37, 43). Some patients with active TB may even recover in the absence of treatment (44). Thus, simple comparison of LTBI and sputum-positive TB may not allow unraveling immune responses leading to TB of variable degrees.

In this study, we analyzed frequencies, numbers, and polyfunctional profile of *Mtb*-specific CD4^+^ cells as well as composition of the general leukocyte population in TBP with different levels of TB severity by scoring TB manifestations. Specifically, we measured the degree of pulmonary destruction, disease extent, bacteria excretion, clinical TB severity, clinical TB forms (see Materials and Methods) and an integrated parameter of pulmonary TB pathology, “Timika X-ray score” (45, 46). By analyzing 27 different quantitative parameters of *Mtb*-specific CD4^+^ T cells in 50 patients with active TB, we found that there were only two statistically significant correlations between magnitude of *Mtb*-specific cytokine-producing CD4^+^ T cells and any of the five TB manifestations. Specifically, the degree of pulmonary destruction correlated with the proportion of TNF-α^+^IFN-γ^+^IL-2^+^ CD4^+^ cells (ρ = 0.37, *p* = 0.008), and the level of bacteria excretion correlated with the numbers of TNF-α^−^IFNg^+^IL-2^+^ CD4^+^ cells (ρ = −0.41, *p* = 0.003). Both associations disappeared after bootstrapping. This strongly suggests that *Mtb*-specific T-cell response plays a minimal role in determining/controlling TB severity. Pulmonary destruction, one of the leading causes of *Mtb* excretion, infection dissemination, and patient’s death, appeared to be most strongly associated with the relative expansion of neutrophils, particularly, band neutrophils, and a decrease in lymphocyte:neutrophil ratios. The results suggest that quantitative parameters of antigen-specific Th1 response play a minor role in determining TB severity, while general shifts in granulocytic and lymphocytic lineages represent an important factor of TB pathogenesis.

## Materials and Methods

### TBP and Evaluation of Disease Manifestations

Fifty HIV-seronegative TBP were recruited from CTRI (Table 1). All patients had recent TB and had not received antituberculosis therapy before the admission to CTRI. The diagnosis was based on clinical and radiological evidences of TB with either identification of *Mtb* and/or *Mtb* DNA in the sputum (*n* = 41) or positive clinical and radiological responses to anti-TB therapy (*n* = 9; the response was assessed 2 months following the treatment by independent clinicians and radiologists unaware of immunological results).

**Table 1:** Study population.

Characteristics	TB patients	TB contacts	Latently infected individuals	Healthy donors
Total number	50	34	13	12
Age, years, median (range)	29 (18–63)	39 (22–62)	45 (24–73)	33 (23–55)
Female, numbers (%)	29 (58%)	28 (82%)	9 (69%)	7 (58%)
TB forms, numbers (%)				
Tuberculoma	5 (10%)	N/A	N/A	N/A
TB infiltrate	32 (64%)	N/A	N/A	N/A
Cavitary TB	6 (12%)	N/A	N/A	N/A
Disseminated TB	7 (14%)	N/A	N/A	N/A
Positive QFT results, numbers (%)	44 (88%)	0	13 (100%)	0

*N/A, not applicable; TB, tuberculosis*.

Five different manifestations of TB were evaluated, i.e., the degree of pulmonary destruction (“Destruction,” notation used in the data file, statistical analyses, and illustrations), TB extent (“TB extent”), the level of bacteria excretion (“Bacteria excretion”), clinical TB severity (“Clinical severity”), and clinical TB forms (“TB forms”). Severity of each TB manifestation was evaluated using TB scoring system developed by independent clinicians, radiologists, and microbiologists before the initiation of the study (Table 2).

**Table 2:** Scoring system used to evaluate severity of TB manifestations.

TB (tuberculosis) manifestations	Parameters evaluated			Final score
Destruction	Number and size of transparent foci:			
	None			1
	One small focus (<2 cm in diameter)			2
	Several small or one large (≥2 cm) focus			3
	Several large foci			4

TB extent	Lung area affected by TB lesions:			
	One-three segments in different lobes			1
	Four or more segments in different lobes or one whole lobe			2
	Two-three lobes in different lungs			3
	One whole lung or both lungs			4

Clinical severity	Body temperature:	Other symptoms:^a^		
	Normal	−		1
	Normal	+		2
	Sub-febrile	−		2
	Sub-febrile	+		3
	Febrile	+		4

Bacteria excretion	*Mtb* counts:^b^	BACTEC results:	PCR results:	
	No or 1–2/30 fields	−	−	1
	No or 1–2/30 fields	− or +	+	2
	1–9/10 fields	+	+	3
	1–9/field	+	+	4
	10–90 or >90/field	+	+	5

TB forms^c^	Tuberculoma			1
	TB infiltrate			2
	Cavitary TB			3
	Disseminated TB			4

*TB patients were examined by independent clinicians, radiologists, and microbiologists; severity of TB manifestations was assessed and scored as indicated in the Table*.

*^a^Cough, weight loss, fatigue, and night sweats*.

*^b^Identified by fluorescence microscopy of auramine-rhodamine-stained sputum*.

*^c^Identified according to the classification of pulmonary TB accepted in Russia*.

The degree of pulmonary destruction, TB extent, and TB forms were evaluated by radiologists based on the results of X-ray and/or computer tomography. TB forms were classified according to TB classification accepted in Russia (Instruction no 109/21.03.2003, Ministry of Health of the Russian Federation). The classification distinguishes more than 10 different forms of pulmonary TB based on clinical and morphological criteria (called “clinical TB forms”), including tuberculoma, TB infiltrate, cavitary TB, disseminated TB, tuberculous pleurisy, cirrhotic lung TB, caseous pneumonia et al. TBP enrolled in our study had four different TB forms (see Table 2).

Clinical disease severity was examined by clinicians.

The presence of *Mtb* and *Mtb* loads in the sputum were determined by microbiologists. Briefly, sputum specimen were decontaminated, digested with standard *N*-acetyl-l-cysteine/sodium hydroxide (BBL MycoPrep; Becton Dickinson, Sparks, MD, USA) and analyzed in auramine-rhodamine smear microscopy, BACTEC MGIT 960 system (BD Diagnostics, Sparks, USA) and real-time IS6110-based PCR (Amplitub-Rv, Syntol, Russia). All procedures were performed according to the manufacturer recommendations.

All TB manifestations were scored as described in Table 2 by radiologists, clinicians, and microbiologists blinded of the results of immunological analyses.

Besides evaluating separate TB manifestations, we also measured “Timika X-ray score,” an integrative parameter of pulmonary pathology. The score was determined as described previously by Kriel et al. and Ralph et al. (45, 46) with small modifications. Briefly, full-size posteroanterior chest X-rays (CXR) were analyzed by two independent radiologists using software Synedra View Personal 3 (version 3.2.0.0; Synedra Information Technologies GmbH, Innsbruck, Austria) and AxioVision LE (Axio Vs40 V 4.8.2.0.; Carl Zeiss AG, Oberkochen, Germany). In Synedra View Personal 3 software, CXR were examined to identify, localize, and characterize TB lesions. If there were discrepancies regarding lesion areas, types or the presence of cavities, they were discussed. After the consensus had been reached, the percentages of affected areas were quantified in AxioVision LE software. To simplify the analysis, the radiologists divided CXR into six zones by outlining the visible zone of the lung and drawing demarcation lines at the top of the anterior edge of the second rib and the bottom of the anterior edge of the forth rib (Figure S1A in Supplementary Material). The area of each zone was determined and recorded. The images were then read from top to bottom to identify TB lesions. For each lesion, the type of opacification (nodular or homogeneous), the presence of lucent foci, the involvement of lymph nodes and pleura were determined; the lesions were outlined, and their areas were determined and recorded. According to the methods previously described (45, 46), areas of lesions with mainly nodular opacification were multiplied by 0.5; similarly, areas of lesions with mainly homogeneous opacification or exudates were multiplied by 1. Areas with the involvement of lymph nodes were multiplied by 0.5. This product for all zones was added and divided by the total visible area of the lungs to determine the percentage of affected lung field. The final “Timika X-ray score” was determined by adding a constant value of 40 if at least one cavity (hypodense area) was identified. Evaluation of “Timika X-ray scores” was performed retrospectively when revising the manuscript in response to reviewers’ comments; therefore, only those patients for whom original X-rays were available, were included in the analysis (*n* = 46 out of 50).

### Healthy Participants

Healthy CTRI employees (*n* = 45) working in tight contacts with TBP for at least 1 year and having no clinical and radiographic evidences of TB were enrolled in the study as individuals with high risk of being infected. Thirty-four of them had negative results of QuantiFERON^®^-TB Gold In-Tube (QFT) assay and formed TB contacts (TBC) group (Table 1).

BCG-vaccinated donors with no records of *Mtb* exposure were enrolled as healthy participants (*n* = 14). Twelve donors had negative QFT results and formed healthy donor group (Table 1). TBC (*n* = 11) and healthy participants (*n* = 2) with positive QFT results formed LTBI group (*n* = 13).

### Immunological Analyses

Immunological analyses were performed within 2 weeks of patient’s admission to CTRI. Blood was collected in heparinized vacutainer tube and used for (i) hematology test; (ii) QuantiFERON^®^-TB Gold In-Tube assay (QFT); (iii) determination of main lymphocyte populations; and (iv) evaluation of *Mtb*-specific CD4^+^ cells producing TNF-α, IFN-γ and/or IL-2.

Main leukocyte populations (i.e., total leukocyte counts, bulk lymphocytes, and segmented and band neutrophils) were determined in hematology test (Beckman Coulter, Brea, USA); QFT was performed according to the manufacture recommendations (Cellestis Ltd, Carnegie, Australia, and Qiagen, Venlo, Netherlands).

Lymphocyte subsets were determined using BD Multitest™ 6-color TBNK reagent containing FITC-anti-CD3, PE-anti-CD16, PE-anti-CD56, Per-CP-Cy5.5-anti-CD45, PE-Cy7-anti-CD4, APC-anti-CD19, and APC-Cy7-anti-CD8 monoclonal antibodies (BD Biosciences, San Jose, USA).

To identify *Mtb*-specific cytokine-producing cells, whole blood was preincubated with PPD (10 μg/ml, 3 h; Accurate Chemical, New York, NY, USA) and then cultured in the presence of Brefeldin A (*GolgiPlug*™; BD Biosciences) for additional 12 h. The cells were stained with PerCP-Cy5.5-anti-CD4 and APC-Cy7-anti-CD8, treated with BD FACS Lysing solution and BD FACS Permeabilizing solution II, and stained with FITC-anti-IFN-γ, APC-anti-TNF-α, and Bv510-anti-IL-2 (BD Biosciences). Cells were analyzed on BD FACSCANTOII flow cytometer equipped with three lasers (BD Biosciences) using BD FASC Diva (BD Biosciences) and FlowJo software (Ashland, OR, USA). Single-stained samples and unstimulated cells were used as controls. During the analysis, cytokine-producing cells were identified within CD4^+^CD8^−^ and CD4^−^CD8^+^ populations. Within CD4^−^CD8^+^ population, less than 0.1% of cells produced IFN-γ, TNF-α, and/or IL-2. Therefore, only CD4^+^CD8^−^ are described in the Results section. The full list of immunological parameters analyzed in the study is presented in Table S1 in Supplementary Material.

### Statistical Analysis

Data are presented as medians and interquartile ranges. Differences between two groups were analyzed using Mann–Whitney test (R, www.r-project.org, FOAS, USA). Differences between several groups were analyzed using one-way analysis of variance in Kruskal–Wallis test with Dunn’s post-test (GraphPad Software, Inc. and/or R). Relationship between TB manifestations and immune response was determined using Spearman rank correlation with Benjamini–Hochberg adjusted method with the false discovery rate *q* = 0.05 (47). We also performed hierarchical clustering of the data using *hclust* routine in R with the distance measure defined as $\log(10^{-5}+p)$ where *p* is the *p*-value from the correlation between two measures (Spearman rank) and a constant 10^−5^ was added to prevent infinite values for correlations reaching extremely low *p*-values. The value of this constant did not influence the results as long as the constant was sufficiently small. Robustness of clustering was accessed by resampling the data (i.e., patients) with replacement 1,000 times (bootstrap analysis). Statistical significance of consistency of clustering of different parameters in bootstrap analysis was accessed by repeating the resampling procedure for randomized data. Some populations of immune cells were associated with multiple manifestations of TB severity. To identify the main predictors for these immune cells, data were normalized using quantile normalization and multiple regression analysis was performed using routine *lm* in R. Minimal regression model was selected using Akaike Information Criteria with routine *step* in R.

## Results

### TBP Do Not Exhibit Deficiency in *Mtb*-Specific Th1 Cells

Previous studies have indicated that *Mtb*-specific CD4 T-cell responses differ between TBP and subjects with LTBI, although data on the character of the differences are not uniform (18–22). To immunologically characterize TBP included in our study, we compared Th1 responses in TBP, individuals with LTBI, TBC, and HD groups. To characterize *Mtb*-specific cytokine-producing CD4^+^ cells, we determined the following parameters:

(i) the frequency of cells producing IFN-γ (IFN-γ^+^), TNF-α (TNF-α^+^), and IL-2 (IL-2^+^) (determined as the percent of IFN-γ^+^, TNF-α^+^, and IL-2^+^ cells out of all CD4^+^ lymphocytes);
(ii) the frequency of CD4^+^ polyfunctional subpopulations (i.e., the percent of TNF-α^+^IFN-γ^−^IL-2^−^, TNF-α^+^IFN-γ^−^IL-2^+^, TNF-α^+^IFN-γ^+^IL-2^−^, TNF-α^+^IFN-γ^+^IL-2^+^, TNF-α^−^IFN-γ^+^IL-2^−^, TNF-α^−^IFN-γ^+^IL-2^+^, and TNF-α^−^IFN-γ^−^IL-2^+^ cells out of all CD4^+^ lymphocytes);
(iii) the frequency of all *Mtb*-specific cells (i.e., the total percent of *Mtb*-responding cells out of CD4^+^ lymphocytes, calculated as the sum of the frequencies of CD4^+^ polyfunctional subpopulations);
(iv) the proportion of CD4^+^ polyfunctional subpopulations, i.e., the percent of CD4^+^ polyfunctional subpopulations out of all *Mtb*-specific cells identified in the study;
(v) the numbers of each subpopulation in the blood (cells/μl).

**Figure 1:**
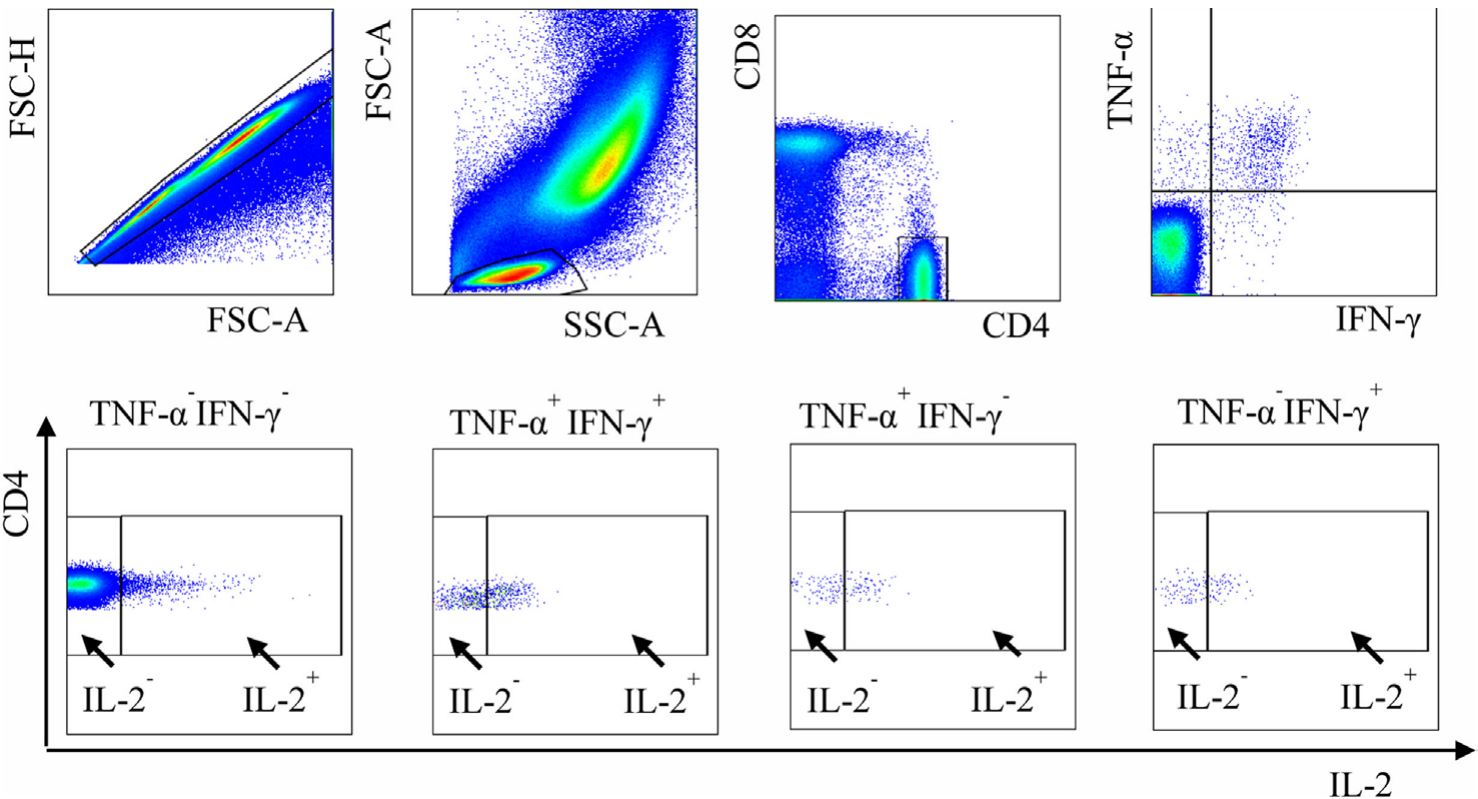
Gating strategy used to define functional subpopulations of *Mycobacterium tuberculosis* (*Mtb*)-specific CD4^+^ cells. Blood cells were stimulated with PPD in the presence of brefeldin A, stained for CD4 and CD8, permeabilized, and treated with mAb specific to IFN-γ, TNF-α, and IL-2. Functional subpopulations of CD4^+^ lymphocytes were defined by flow cytometry after gating sequentially on singlets, lymphocytes, CD4^+^ cells, and IFN-γ/TNF-α- and IL-2-producing cells.

The strategy of cell identification is shown in Figure 1; the full list of analyzed parameters is presented in Table S1 in Supplementary Material. The usage of several approaches to evaluate functional subpopulations was needed because different studies utilized different methodologies to quantify these populations [e.g., determined cell percent out of antigen-specific (27) or out of all (31) CD4^+^ T cells].

**Figure 2:**
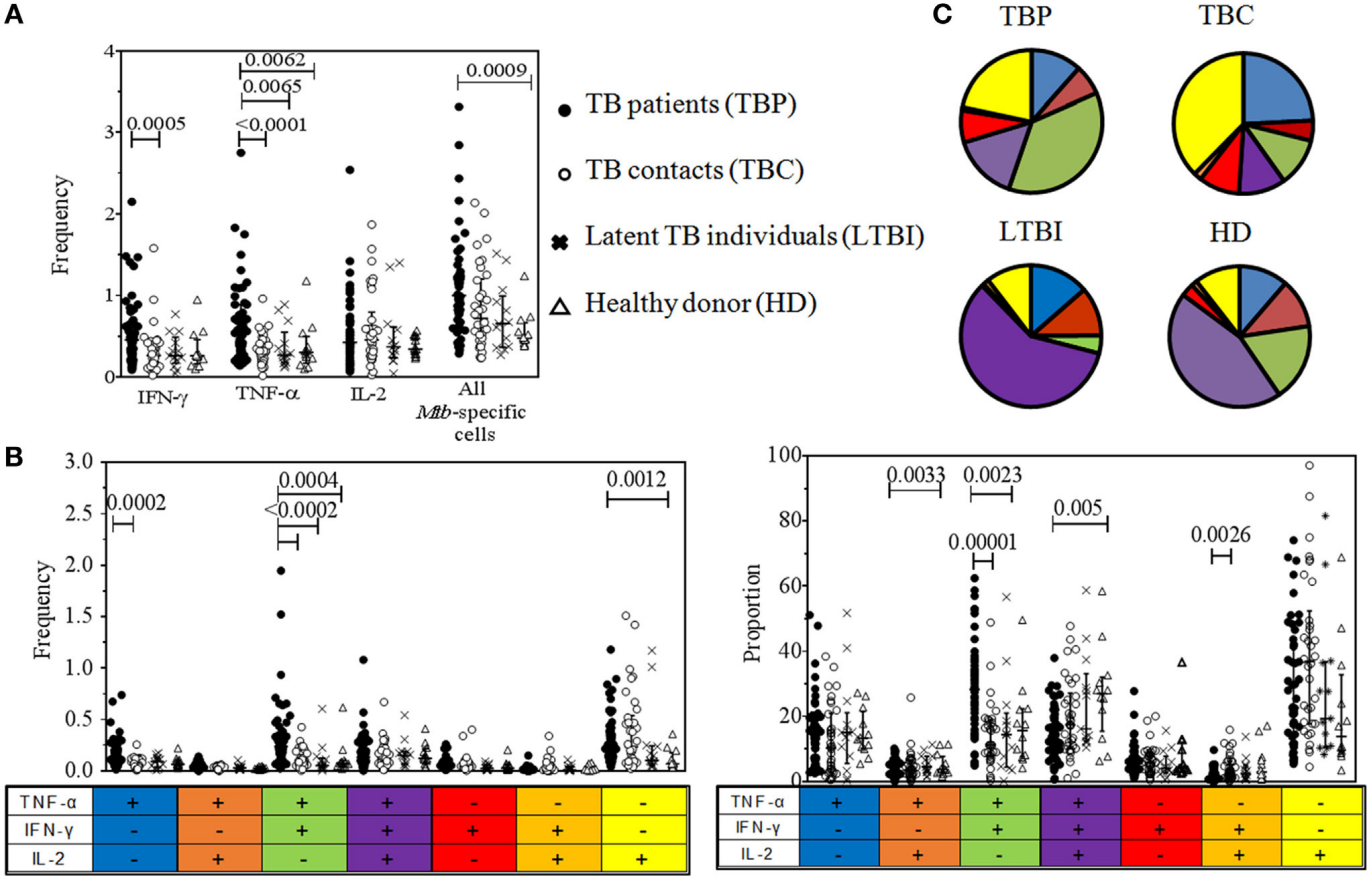
Quantitative analysis of cytokine-producing CD4^+^ lymphocytes in tuberculosis (TB) patients and control groups. Blood cells were stimulated with PPD and CD4^+^ cells containing intracellular IFN-γ, TNF-α, and/or IL-2 were identified by flow cytometry. **(A)** Frequencies of IFN-γ^+^, TNF-α^+^, IL-2^+^, and all *Mycobacterium tuberculosis* (*Mtb*)-specific cells (percent after gating on CD4^+^ lymphocytes). **(B)** Frequencies of polyfunctional subpopulations of *Mtb*-specific CD4^+^ cells (percent after gating on CD4^+^ lymphocytes). **(C)** Proportions of polyfunctional subpopulations of *Mtb*-specific CD4^+^ cells, shown in pie charts and the graph (proportion out of all *Mtb*-specific CD4^+^ cells). Data were analyzed using Kruskal–Wallis test with Dunn’s post-test. False discovery rate was set at *q* = 0.05. The cutoff *p*-values were 0.0104 for data shown in **(A)**, 0.006 for data shown in **(B)**, and 0.0071 for data shown in **(C)**. Note that numbers of comparisons were different for data shown in **(A)** and **(B,C)**. For **(A)**: *n* = 24 (four immunological parameters compared in four groups of participants, i.e., six intergroup comparisons for each of four immunological parameters); for **(B,C)**: *n* = 42 (7 immunological parameters compared in four groups of participants, i.e., six intergroup comparisons for each of seven immunological parameters). Only significant differences are shown.

Tuberculosis patients had higher frequencies of TNF-α^+^ cells compared to all healthy groups, higher frequencies of IFN-γ^+^-producing cells compared to TBC, and higher frequencies of all *Mtb*-specific cells compared to HD (Figure 2A). Individual variability in cytokine-producing CD4^+^ cells was higher in TBP than in other groups.

The polyfunctional profile of *Mtb*-specific CD4^+^ cells differed in TBP and other groups (Figures 2B,C). Particularly, TBP had (i) higher frequencies (*p* < 0.0005) of bifunctional TNF-α^+^IFN-γ^+^IL-2^−^ lymphocytes compared to all healthy groups and higher proportions of these cells compared to TBC and HDs (*p* < 0.005); (ii) higher frequencies of monofunctional TNF-α^+^IFN-γ^−^IL-2^−^ lymphocytes compared to TBC (*p* = 0.0002) and monofunctional TNF-α^−^IFN-γ^−^IL-2^+^ lymphocytes compared to HD (*p* < 0.002); (iii) lower proportions of trifunctional TNF-α^+^IFN-γ^+^IL-2^+^ lymphocytes compared to HD (*p* = 0.005); and (iv) lower proportions of bifunctional TNF-α^−^IFN-γ^+^IL-2^+^ and TNF-α^+^IFN-γ^−^IL-2^+^ lymphocytes compared to TBC and HD (*p* < 0.005).

Thus, TBP did not exhibit deficiency and even had elevated levels of *Mtb*-specific CD4^+^ cells producing type 1 cytokines. Functional profile of *Mtb*-specific CD4^+^ cells in TBP was biased out of polyfunctional activity toward the accumulation of bifunctional TNF-α^+^IFN-γ^+^ lymphocytes lacking IL-2 production, which is in line with the results of several other studies (28, 29, 48).

### Variable Correlations between Different TB Manifestations

Our analysis revealed a great variability of frequencies, numbers, and polyfunctionality of *Mtb*-specific CD4^+^ cells in TBP. It was possible that this variability was driven by variable aspects of TB manifestations and/or underlied these aspects. To examine associations between immune responses and TB manifestations, five distinct manifestations of TB were evaluated by independent experts in each enrolled patient (Table 2): pulmonary destruction, TB extent, clinical severity, bacteria excretion, and TB forms (see Materials and Methods). It is important to emphasize that each TB manifestation was evaluated and scored blindly by clinicians, radiologists, and microbiologists. We hypothesized that if different manifestations of TB similarly measure TB severity, there should be strong correlations between these manifestations.

**Figure 3:**
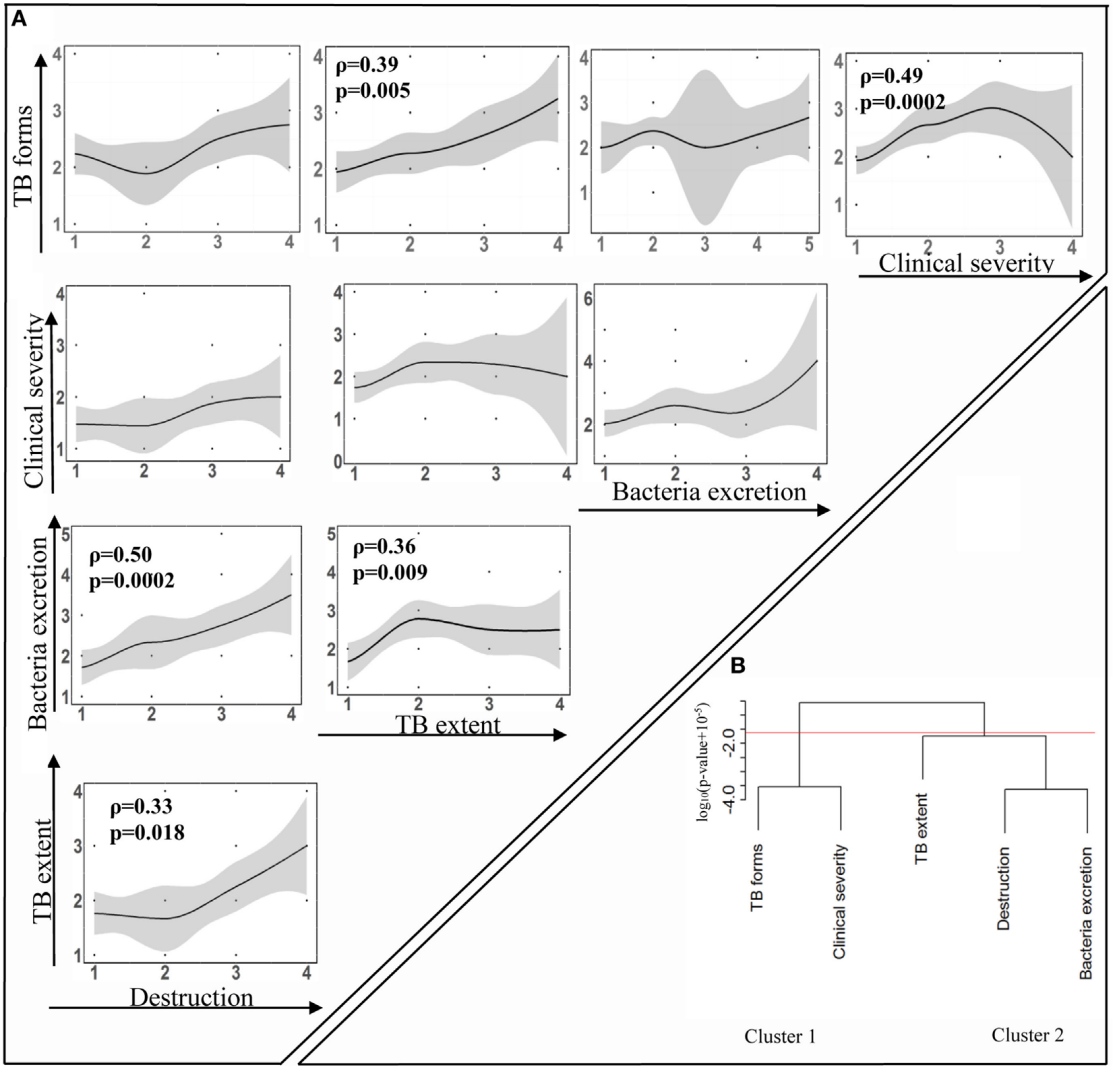
Relationships between diverse tuberculosis (TB) manifestations. TB patients were examined to evaluate the following five TB manifestations: the degree of pulmonary destruction (“Destruction”), TB extent, the level of bacteria excretion (“Bacteria excretion”), clinical TB severity (“Clinical severity”), and clinical TB forms (“TB forms”). Each TB manifestation was scored (Table 2), and correlations between them were analyzed using Spearman analysis with FDR adjusted method. To identify significant correlations and avoid type one error, false discovery rate was set at *q* = 0.05 and the significance threshold for correlations between five measured parameters was *p* = 0.025. **(A)** Correlations are visualized by showing best-fit lines and scatter plots. Indicated are correlation coefficient ρ and adjusted *p*-values. Figures on the axes indicate scores of corresponding TB manifestation. **(B)** Cluster tree. Hierarchical clustering of the data was performed using *hclust* routine in R (see Materials and Methods). FDR defined cutoff for significance of *p* = 0.025 led to the division of correlations into two major clusters.

Despite our expectations, our analysis showed that not all measured manifestations correlated significantly with each other (Figure 3A). TB forms correlated with TB extent and clinical severity (*p* = 0.005 and *p* = 0.0002, respectively). Destruction and TB extent correlated with bacteria excretion (*p* = 0.0002 and *p* = 0.009, respectively) and with each other (*p* = 0.018). However, clinical severity did not associate with the destruction, bacteria excretion, or TB extent.

Hierarchical clustering led to similar interpretation of significant correlations with two major clusters defined at the *p* = 0.025 cutoff level: cluster 1, TB forms, and clinical severity (Figure 3B, cluster 1), and cluster 2, destruction, bacteria excretion, and TB extent (Figure 3B, cluster 2). Resampling with replacement of data for individual patients and clustering of resampled data revealed stability of cluster 1 in nearly 80% of simulations, while only destruction and bacteria excretion clustered reliably in cluster 2 (about 80% of simulations).

### Patients with Different Levels of TB Severity Did Not Differ Substantially by the Quantities or Polyfunctional Profile of *Mtb*-Specific CD4^+^ Cells But Exhibited Highly Significant Differences in Neutrophil and Bulk Lymphocyte Populations

**Figure 4:**
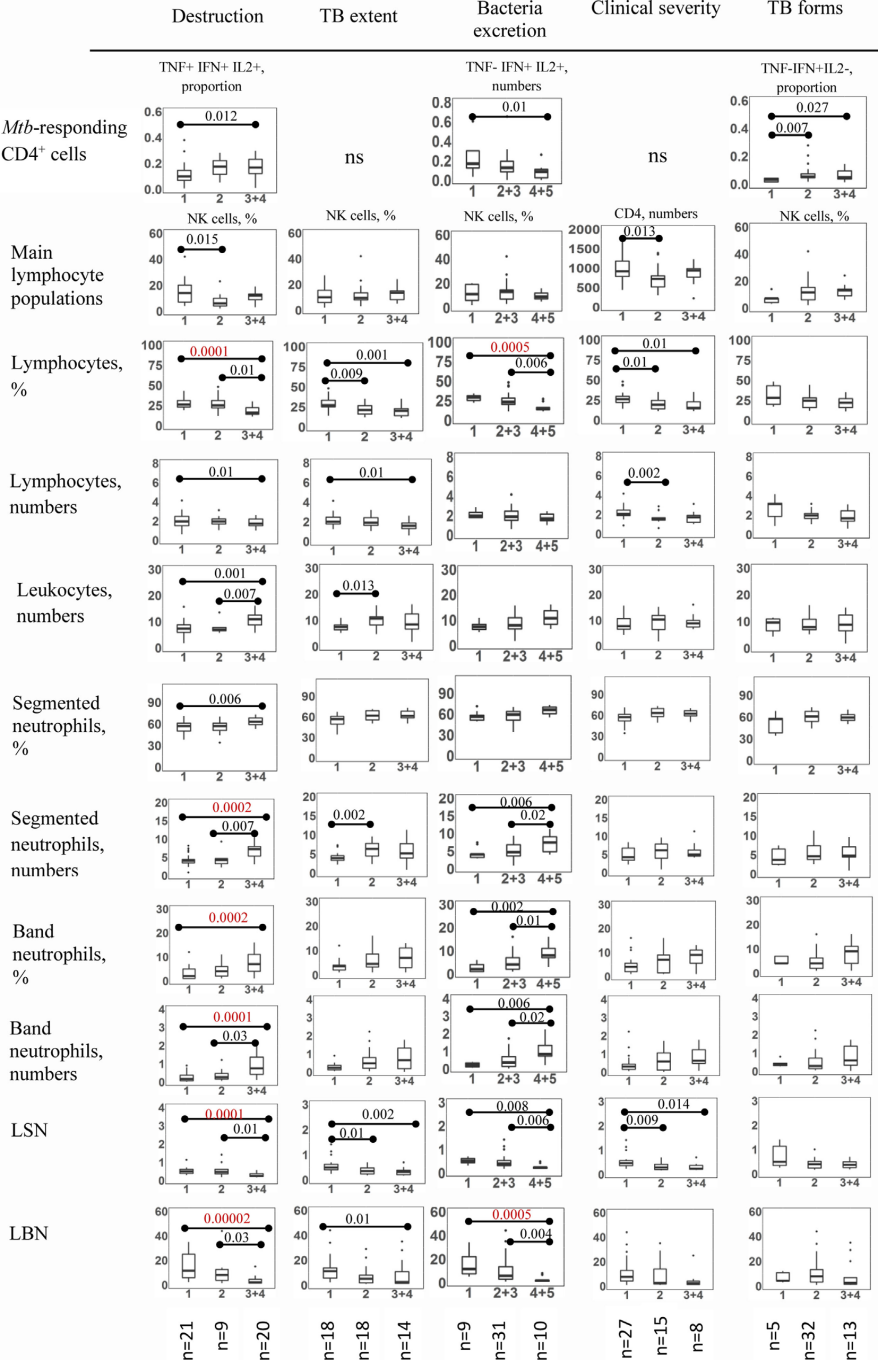
Immunological differences between TB patients (TBP) exhibiting different severities of tuberculosis (TB) manifestations. TBP were grouped based on the severity of particular TB manifestation (scored as described in Table 2). To simplify the analysis and compensate for a relatively low number of TBP in some groups, some groups of TBP were combined as indicated in the figure (i.e., TBP having scores 2 and 3 for bacteria excretion; 4 and 5 for bacteria excretion; and 3 and 4 for other TB manifestations). Immunological differences between the groups were analyzed using Kruskal–Wallis test with Dunn’s post-test (R). False discovery rate was set at *q* = 0.05. The significance thresholds were determined in the following two ways: (i) separately for each comparison, the number of tests set on 3 (for three groups of comparison; for different comparisons, the *p*-value cutoff varied from 0.0167 to 0.033); (ii) for all types of comparisons, the number of tests set on 615 (for 41 immunological parameters, five TB severity manifestations, three groups of comparison; for all comparisons, the cutoff *p*-value = 0.00065). Figures indicate significant differences for *N* = 3. Differences that were significant after applying FDR correction for *N* = 615 are highlighted in red. Immunological parameters that did not differ significantly between the groups are not shown. Figures on *X*-axis indicate scores of the corresponding TB manifestation. Cell numbers are shown in cells/μl. Numbers of patients in each group are shown at the bottom of the figure.

Division of five different TB manifestations into two independent clusters could arise due to several reasons. First, it was possible that measurement of these TB manifestations was noisy, and thus, clustering was observed due to chance. A larger cohort will be needed to test this specific hypothesis because resampling showed some robustness in cluster structure (see above). Alternatively, different TB manifestations could arise due to different arms of the immune responses. To address, this hypothesis that we investigated whether particular TB manifestations had specific immunological signatures. For that, we divided TBP into groups with different severity of a given TB manifestation and analyzed all immunological parameters examined in the study in these groups. To simplify the analysis and compensate for a relatively low number of TBP in some groups (usually, having more severe TB), TBP having highest severity scores were combined in one group (i.e., TBP having bacteria excretion scores 4 and 5 and TBP having scores 3 and 4 for all other TB manifestations). TBP having scores 2 and 3 of bacteria excretion were also combined. This allowed comparing immunological parameters in TBP having the least, the highest and medium degrees of each of five TB manifestations (Figure 4). The differences between the groups were analyzed using Kruskal–Wallis test with Dunn’s post-test (R) and false discovery rate set at *q* = 0.05. The significance thresholds were determined in the following two ways: (i) separately for each comparison (three groups of comparison; for different comparisons, the *p*-value cutoff varied from 0.0167 to 0.033), and (ii) for all types of comparisons (the number of tests set on 615, the cutoff *p*-value = 0.00065, see Legend to Figure 4 for details).

The frequencies of *Mtb*-specific IFN-γ^+^, TNF-α^+^, or IL-2^+^ cells did not differ between the groups. The differences in polyfunctional populations were seen only when the threshold of significance was set for three groups of comparison and were insignificant when a threshold of *p* = 0.00065 was applied (Figure 4). Particularly, the proportion of TNF-α^+^IFN-γ^+^IL-2^+^ cells was higher in TBP with high pulmonary destruction compared to TBP without destruction (*p* = 0.01); the numbers of TNF-α^−^IFN-γ^+^IL-2^+^ CD4^+^ cells were higher in TBP without bacteria excretion compared to TBP with high level bacteria excretion (*p* = 0.01); the proportion of TNF-α^−^IFN-γ^+^IL-2^−^ cells was lower in patients with tuberculoma compared to TBP with TB infiltrate (*p* = 0.007) and cavitary/disseminated TB (*p* = 0.03, Figure 4).

Similarly, analysis of main lymphocyte populations (i.e., CD4^+^, CD8^+^, CD19^+^, and CD16/CD56^+^) revealed some differences between the groups only when the significance threshold was set for three groups of comparison. In this analysis, the frequency and the numbers of CD16/CD56^+^ cells were higher in TBP with no destruction compared to TBP with medium degree of pulmonary destruction (*p* = 0.02 and *p* = 0.01, Figure 4 and data not shown); the numbers of CD4^+^ cells were higher in TBP with low clinical TB severity compared to TBP with medium degree of clinical TB severity (*p* = 0.01, Figure 4). The differences were not significant at the threshold set at *p* = 0.00065.

In contrast to *Mtb*-specific CD4^+^ cells and main lymphocyte populations, leukocyte counts, neutrophil, and bulk lymphocyte populations differed significantly between the groups. The most significant differences were registered between groups of patients having different degrees of pulmonary destruction and different levels of bacteria excretion. Specifically, TBP with high pulmonary destruction had lower percentages and numbers of lymphocytes (*p* = 0.0001 and *p* = 0.01, respectively), higher leukocyte numbers (*p* = 0.001), higher percentages and numbers of segmented neutrophils (*p* = 0.006 and *p* = 0.0002, respectively), and band neutrophils (*p* = 0.0002 and *p* = 0.0001, respectively, Figure 4). The lymphocyte:segmented neutrophils (LSN) and lymphocyte:band neutrophils (LBN) ratios were dramatically decreased in patients with severe destruction (2.5- and 4.5-fold compared to TBP without destruction, *p* = 0.0001 and *p* = 0.00002, respectively). Of note, most of the differences had *p*-value < 0.00065 (Figure 4). Similar pattern of differences was characteristic for TBP exhibiting different levels of bacteria excretion, though the significance levels were generally lower (Figure 4).

Tuberculosis patients with different TB extent differed by the percentages and numbers of lymphocytes (higher in TBP with less TB extent, *p* = 0.001 and *p* = 0.01, respectively), the numbers of leukocytes and segmented neutrophils (higher in TBP with higher TB extent, *p* = 0.013 and *p* = 0.002, respectively), LSN, and LBN (higher in TBP with less TB extent, *p* = 0.002 and *p* = 0.01, respectively). Compared to pulmonary destruction, the significance of the differences was lower, band neutrophils were not associated with TB extent, and the differences were insignificant for *p* = 0.00065 threshold (Figure 4).

Clinical TB severity was not associated with neutrophil populations. TBP with severe, medium, and mild degrees of clinical disease severity differed only by bulk lymphocyte population (higher in TBP with less severe TB, *p* = 0.01 and *p* = 0.002 for lymphocyte percent and numbers, insignificant for *p* = 0.00065 threshold; Figure 4).

Overall, we found no consistent associations between the severity of TB manifestations and the quantities or polyfunctional profile of circulating *Mtb*-specific CD4^+^ cells. In contrast, the circulating segmented and band neutrophils and bulk lymphocyte population associated strongly with the severity of several particular TB manifestations, primarily – with pulmonary destruction and bacteria excretion. To further examine these associations, we performed correlation analysis.

### Correlation Analysis Identifies Leukocyte Subsets, in Particular Band Neutrophils, As Strong Correlates of Destructive Pulmonary TB

We next calculated correlations between the magnitude of each TB manifestation and immunological parameters. In total, we analyzed correlations between 46 measured parameters in 50 patients. False discovery rate was set at *q* = 0.05, and for this analysis, threshold was *p* = 0.0097 (Table 3). Interestingly, in this analysis, there were only two statistically significant correlations between *Mtb*-specific CD4^+^ populations and TB manifestations, i.e., the proportion of TNF-α^+^IFN-γ^+^IL-2^+^ CD4^+^ cells correlated positively with pulmonary destruction (*p* = 0.008) and the numbers of TNF-α^−^IFNg^+^IL-2^+^ CD4^+^ cells correlated negatively with bacteria excretion (*p* = 0.003). Other populations of *Mtb*-specific CD4^+^ cells did not correlate with TB manifestations.

**Table 3:** Correlations between severity of tuberculosis (TB) manifestations and immunological parameters.

Variables	Destruction, rho (*p*-value)	Bacteria excretion, rho (*p*-value)	TB extent, rho (*p*-value)	Clinical severity, rho (*p*-value)
TNF-α^+^IFN-γ^+^IL-2^+^, proportion	0.369 (0.00790)	ns	ns	ns
TNF-α^−^IFN-γ^+^IL-2^+^, numbers	ns	−0.405 (0.00324)	ns	ns
Leukocytes, count	0.442 (0.00115)	0.379 (0.00621)	ns	ns
Segmented neutrophils, percent	0.373 (0.00725)	ns	ns	ns
Segmented neutrophils, number	0.509 (0.00012)	0.433 (0.0015)	ns	ns
Band neutrophils, percent	**0.562 (1.3 × 10**^−^**^5^)**	0.442 (0.00113)	ns	ns
Band neutrophils, number	**0.580 (5.5 × 10**^−^**^6^)**	0.469 (0.00048)	ns	ns
Lymphocytes, percent	−**0.562 (1.3 × 10**^−^**^5^)**	−0.504 (0.00014)	−0.510 (0.00011)	−0.447 (0.00098)
Lymphocytes, number	ns	ns	ns	−0.449 (0.00092)
LSN	**0.551 (2.2 × 10**^−^**^5^)**	−0.472 (0.00044)	−0.492 (0.00023)	−0.447 (0.00097)
LBN	−**0.633 (3.1 × 10**^−^**^7^)**	−0.502 (0.00015)	−0.413 (0.00261)	ns

*Blood cells obtained from TB patients were analyzed for: (a) the frequencies and the numbers of *Mtb*-specific IFN-γ^+^ and TNF-α^+^ CD4^+^ cells; (b) the frequencies, proportions, and numbers of polyfunctional subpopulations of *Mtb*-specific CD4^+^ lymphocytes; (c) main lymphocyte populations; and (d) leukocyte counts, neutrophil, and bulk lymphocyte populations (41 immunological variable, Table S1 in Supplementary Material). Each immunological variable was correlated with the severity of five TB manifestations (evaluated and scored as indicated in Table 2). In total, we analyzed correlations between 46 measured parameters. False discovery rate was set at *q* = 0.05, and the significance threshold was *p* = 0.0097. Only significant correlations are shown. Correlations with *p*-value <0.0001 are highlighted in bold*.

In contrast, multiple significant correlations were detected between TB manifestations and leukocyte populations (Table 3). Particularly, destruction correlated directly with leukocyte counts (*p* = 0.001), segmented neutrophils (*p* = 0.007 and *p* = 0.0001 for cell percent and numbers, respectively), and band neutrophils (*p* = 1 × 10^−5^ and *p* = 6 × 10^−6^ for cell percent and numbers, respectively). Correlations between pulmonary destruction, the percent of lymphocytes, LSN, and LBN were inverse (*p* = 1 × 10^−5^, *p* = 2 × 10^−5^, and *p* = 3 × 10^−7^, respectively). Similarly, bacteria excretion correlated directly with leukocyte counts (*p* = 0.006), segmented neutrophils (numbers, *p* = 0.002), band neutrophils (percent and numbers, *p* = 0.001 and *p* = 0.0005, respectively), and inversely – with lymphocytes (percent), LSN, and LBN (*p* = 0.0001, *p* = 0.0004, and *p* = 0.0002, respectively, Table 3).

Tuberculosis extent and clinical severity did not correlate significantly with neutrophil populations. Correlations were registered between (a) TB extent and lymphocyte percent (*p* = 0.0001), LSN (*p* = 0.0002), and LBN (*p* = 0.003) and (b) clinical severity and lymphocyte percent, numbers, and LSN (*p* = 0.001). For TB forms, no significant correlations were found.

### Hierarchical Clustering Analysis Confirms Insignificance of *Mtb*-Specific CD4^+^ Cells As Associates of TB Severity and the Impact of Neutrophils in Pulmonary Destruction

To confirm and extend our results on correlation between immune response and TB manifestations, we performed hierarchical clustering analysis of all of our 46 measured parameters in 50 patients. The measure of distance between different parameters was significance of the correlation characterized by the *p*-value in Spearman rank test (see also Materials and Methods). The cutoff for clusters was defined for false discovery rate of *q* = 0.05 as *p* = 0.0097.

**Figure 5:**
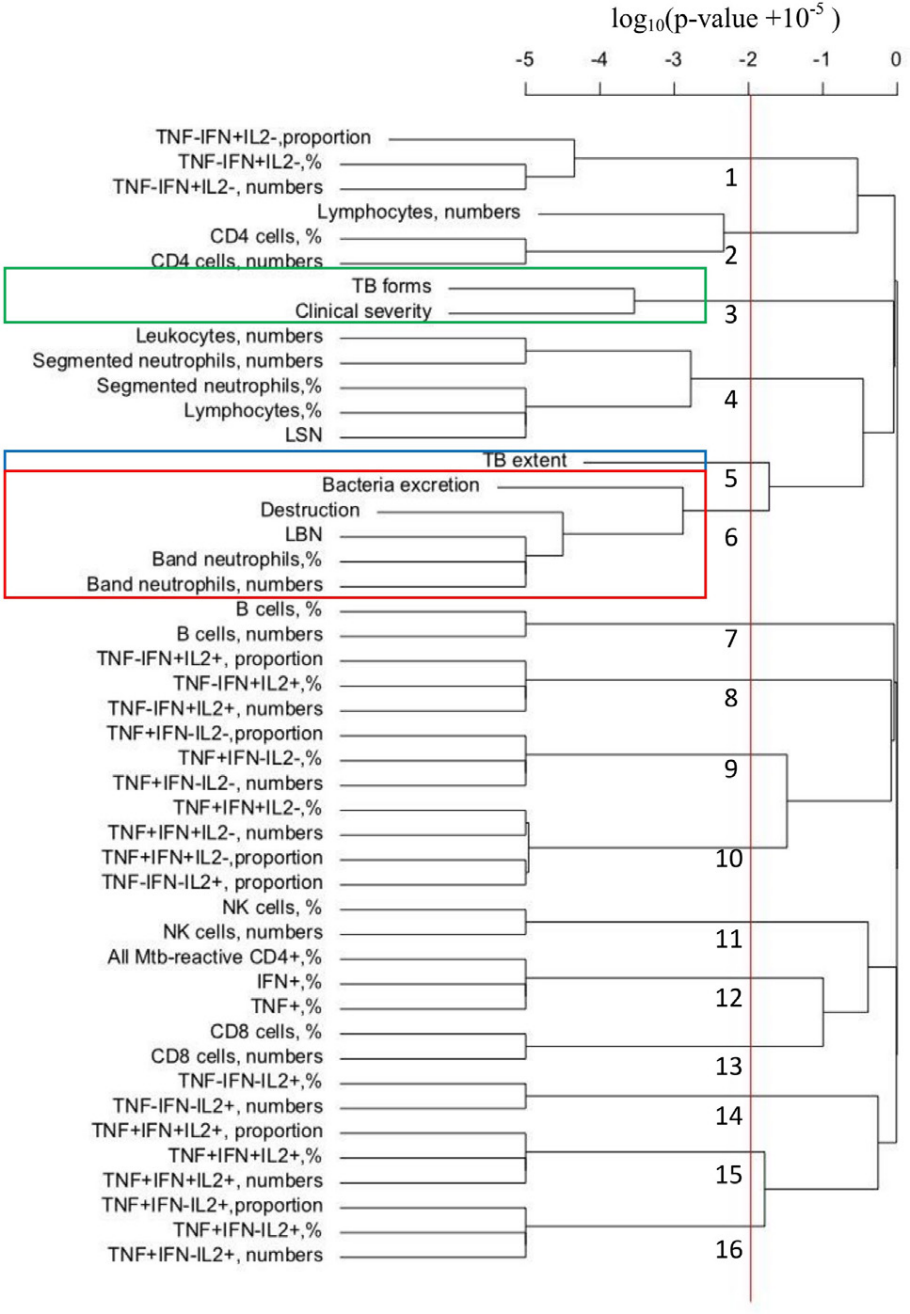
Hierarchical clustering analysis of tuberculosis (TB) manifestations and immunological parameters: *Mycobacterium tuberculosis* (*Mtb*)-specific CD4^+^ cells do not cluster with any TB manifestation, whereas band neutrophils cluster with pulmonary destruction. For each patient included in the study, five TB manifestations were evaluated and scored as shown in Table 2 and 41 immunological parameters were determined (Table S1 in Supplementary Material). Hierarchical clustering of the data was performed using *hclust* routine in R (see Materials and Methods).

Analysis revealed that TB forms, clinical severity, and TB extent did not strongly correlate with any of immunological parameters. In contrast, both destruction and bacteria excretion (previously found to form a cluster, Figure 3B) were strongly correlated with innate response and specifically, band neutrophils (percent, numbers, and LBN, Figure 5).

Interestingly, many immunological measurements were clustered together, for example, percent and total number of B cells, which is expected because total number of a particular lymphocyte population is generally calculated by multiplying percentages by total number of lymphocytes. Other clusters arose because of strong negative correlations, e.g., the correlations between the proportion, frequency, and numbers of TNF-α^+^IFN-γ^+^IL-2^−^ cells and the proportion of TNF-α^−^IFN-γ^−^IL-2^+^ lymphocytes (*p* = 4 × 10^−7^).

Resampling the data with replacement and performing the clustering revealed that the clusters identified by analysis of original data were not fully robust (Table 4). However, clustering of destruction with band neutrophils (as percent out of leukocytes, absolute number, or LBN) was highly reproducible and found in 707 out of 1,000 simulations (frequency rate, 0.707, Table 4). Other associations were weaker although still significant since randomization of the data led to clustering of different measures in at most 88 out 1,000 simulations (we thus set cutoff of significance at as a double of the clustering rate in randomized data, *c* = 0.18). In particular, leukocyte and segmented neutrophil counts clustered with destruction with the frequency rates 0.355 and 0.354, respectively.

**Table 4:** The results of cluster analysis of tuberculosis (TB) manifestations and immunological parameters after bootstrap resampling (frequency rates).

Variable	Destruction	Bacteria excretion	TB extent	Clinical severity	TB forms
Destruction	1	**0.543**	0.128	0.018	0.032
Bacteria excretion	**0.543**	1	0.163	0.022	0.002
TB extent	0.128	0.163	1	0.083	**0.262**
Clinical severity	0.018	0.022	0.083	1	**0.575**

TB forms	0.032	0.002	**0.262**	**0.575**	1
Leukocytes, count	**0.355**	**0.271**	0.068	0.006	0.003
Segmented neutrophils, %	0.135	0.106	0.133	0.133	0.005
Segmented neutrophils, numbers	**0.354**	**0.270**	0.069	0.006	0.003
Band neutrophils, %	**0.707**	**0.497**	0.121	0.012	0.011
Band neutrophils, numbers	**0.707**	**0.497**	0.121	0.012	0.011
Lymphocytes, %	0.159	0.128	0.137	0.130	0.005
Lymphocytes, numbers	0.01	0.008	0.136	**0.317**	0.075
LBN	**0.707**	**0.497**	0.121	0.012	0.011

*Hierarchical clustering analysis was performed for five TB manifestations and all of 41 immunological parameters measured in 50 patients. Cluster analysis with bootstrap resampling (1,000 iterations) was performed in R. Indicated are frequency rates. Only parameters having significant associations are shown. Significant associations are shown in bold (cutoff of significance set at *c* = 0.18)*.

Among other associations, bacteria excretion clustered with band neutrophils (percent, numbers, and LBN; frequency rates, 0.497), leukocyte, and segmented neutrophil numbers (frequency rates, 0.271 and 0.270, respectively); clinical severity was associated with lymphocyte numbers (frequency rate, 0.317). Other immunological parameters including *Mtb*-specific CD4^+^ cells and their functional subpopulations did not cluster significantly with any TB manifestation (frequency rate <0.180).

To further address associations between immunological parameters and TB manifestations, we performed multiple regression analysis and examined which TB manifestations explained best the variances in different immune cells. The following immunological parameters were included in the analysis as dependent variables: frequencies of TNF-α^+^, IFN-γ^+^, and all *Mtb*-specific CD4^+^ lymphocytes; frequencies of seven polyfunctional subpopulations of *Mtb*-specific cells; frequencies of CD4^+^, CD8^+^, CD19^+^, and CD16/CD56^+^ cells; leukocyte count, percentages and numbers of lymphocytes, and segmented and band neutrophils. Independent variables (determinants) were destruction, TB extent, bacteria excretion, clinical severity, and TB forms. Before performing regression analysis, data were normalized, and for each immunological parameter, full and minimal models were generated. The dependence existing between several TB manifestations (see Figure 3B) was taken into account when performing analysis in R.

Minimal models for different *Mtb*-specific populations of CD4^+^ lymphocytes (e.g., the frequency of TNF-α^+^ lymphocytes, TNF-α^+^IFN-γ^−^IL-2^−^ cells, etc.) included multiple parameters, suggesting that these populations did not associate preferentially with any particular TB manifestation (Table S2 in Supplementary Material). Among different TB manifestations, clinical severity was included most often as a significant factor explaining the variances in *Mtb*-specific populations, and it had a positive estimate (except the model for the frequency of TNF-α^+^IFN-γ^+^IL-2^+^ trifunctional cells, which did not include clinical severity). This suggests that an increase in *Mtb*-specific populations rather mirrored clinical severity and/or contributed to it than protected against it.

Similar to the models explaining *Mtb*-specific populations of cells, minimal models for lymphocytes and segmented neutrophils included several different TB manifestations. Of note, minimal models explaining the frequency or the number of lymphocytes included clinical severity and other TB manifestations as negative correlates, suggesting a protective role of lymphocyte population during TB (discussed below). In contrast, in minimal models explaining the accumulation of segmented neutrophils, different TB manifestations were included as positive correlates, likely mirroring the pathological role for neutrophils in TB pathogenesis.

As said earlier, the variances in *Mtb*-specific populations of lymphocytes, bulk lymphocytes, and segmented neutrophils could not be explained well by any particular TB manifestation. In contrast, minimal models for band neutrophils could be explained well only by pulmonary destruction (band neutrophil percent, *p* = 3 × 10^−5^) or by pulmonary destruction in combination with bacteria excretion (*p* = 0.001 and *p* = 0.1, Table S2 in Supplementary Material).

Thus, minimal models for *Mtb*-specific CD4^+^ cells, total neutrophil, and lymphocyte populations differed. The results further confirmed association between pulmonary destruction and band neutrophils and suggested possible link between clinical severity, a deficiency in bulk lymphocyte population and an expansion of several *Mtb*-specific populations (the latter was not fully supported by other types of our analyses).

### Band Neutrophils Correlate with Integrative Parameter of Pulmonary Pathology (Timika X-Ray Score)

The scoring system developed and applied in our study was based on separate evaluation of distinct TB manifestations. Particularly, it discriminated TB extent and the level of pulmonary destruction. However, the system missed an integrative parameter that would evaluate the overall severity of pulmonary pathology and would take into account the complexity of pathological processes ongoing in the lungs during TB. Recently, such integrative parameter “Timika X-ray score” has been described and suggested to evaluate chest X-ray TB severity (45, 46). We therefore examined whether separate TB manifestations measured using our score system were associated with “Timika X-ray score” and how the latter was associated with immunological parameters evaluated in the study.

In correlation and cluster analyses, TB forms and clinical severity did not associate with “Timika X-ray score.” In contrast, TB extent, bacteria excretion, and especially, pulmonary destruction were highly significant correlates of “Timika X-ray score” (*p* = 0.002, *p* = 3 × 10^−5^, and *p* = 1 × 10^−5^, respectively; Figure S1B in Supplementary Material).

When “Timika X-ray score” was included in hierarchical clustering analysis together with other 46 measured parameters (see Figure 5), it clustered primarily with pulmonary destruction and band neutrophils (percent, numbers, and LBN; Figure S1D in Supplementary Material). No significant correlations were found between “Timika X-ray score” and *Mtb*-specific populations of cells (the lowest *p*-value was 0.04 for the proportion of TNF-α^−^IFN-γ^+^IL-2^+^ cells, which was insignificant after applying FDR correction).

To summarize, in different analyses, TB severity was not associated with the quantitative parameters of *Mtb*-specific CD4^+^ responses or polyfunctional profile of *Mtb*-specific CD4^+^ cells. An increase in neutrophil populations and a deficiency in bulk lymphocyte population appeared as the main immunological correlates of TB severity. The main immunological correlate of pulmonary destruction, one of the leading causes of *Mtb* excretion and infection dissemination, was an increase in the percent and absolute number of band neutrophils.

## Discussion

Tuberculosis is an infectious disease, which progression and outcomes critically depend on host immune reactivity. It is assumed that both immune deficiency and hyper-reactivity are involved in TB pathogenesis, but exact immunological correlates of TB protection and pathology and their quantitative measures are not known. In this study, we dissected and quantified several different manifestations of pulmonary TB and analyzed how these manifestations are associated with quantitative parameters of *Mtb*-specific CD4^+^ cells and general leukocyte populations. The results obtained provide an insight in several aspects of TB pathogenesis.

By performing correlative and hierarchical clustering analyses, we document an association between several TB manifestations, i.e., between pulmonary destruction and the level of *Mtb* excretion; between these two manifestations and TB extent; between clinical TB severity and “TB forms” (which in this study included tuberculoma, TB infiltrate, cavitary TB, and disseminated TB, identified according to the classification of pulmonary TB forms accepted in Russia). These associations (e.g., an association between lung tissue destruction and bacilli expel into the environment) are in line with the current understanding of TB pathogenesis (49). However, our analysis also revealed lack of correlation between certain TB manifestations, i.e., between clinical severity and pulmonary destruction, *Mtb* excretion, and TB extent. This finding is new. It may be explained by a “noisy” measurement of TB manifestations, which are ranked on a scale. Differently, the lack of correlation may indicate that clinical TB severity (evaluated on the basis of fever, night swears, fatigue, and other clinical TB symptoms) is not a direct consequence of tissue destruction or *Mtb* spread in the lungs and is underlied by other mechanisms. Immunological analyses performed in the study are consistent with the second hypothesis.

Our immunological analysis was mainly focused on circulating *Mtb*-specific CD4^+^ cells producing type 1 cytokines. Comparison of the frequencies, numbers, and polyfunctional profile of these cells in TBP and healthy participants showed that TBP had more *Mtb*-specific Th1 cells. These results correspond well to several other studies that reported no deficiency in *Mtb*-specific cytokine-producing cells during active TB compared to LTBI. For example, Chiappini et al. showed that children with active TB had increased frequencies of IFN-γ- and IL-2-producing cells specific to *Mtb* antigens AlaDH and TB 10.3 and similar frequencies of cytokine-producing cells specific to several other *Mtb* antigens compared to children with LTBI (23). Given that only patients with recent TB were included in our study, the results demonstrate that there is no deficiency in *Mtb*-specific Th1 populations at the early stages of TB disease. Analysis of polyfunctional populations showed that *Mtb*-specific CD4^+^ cells persisting during active TB were biased out of polyfunctional activity toward the accumulation of TNF-α^+^IFN-γ^+^ bifunctional lymphocytes. These results are in line with several other studies that reported accumulation of bifunctional TNF-α^+^IFN-γ^+^ and monofunctional TNF-α^+^ cells during active TB and associated polyfunctional populations with latency (28, 29, 31, 40). Overall, TBP differed from healthy participants (including LTBI subjects) by the frequencies, numbers, and polyfunctional profile of *Mtb*-specific CD4^+^ cells.

However, our analyses of *Mtb*-specific CD4^+^ cells in TBP did not reveal consistent correlations between cell frequencies, numbers or polyfunctional profile, and severity of TB manifestations. The results can be interpreted in a way that quantitative parameters of *Mtb*-specific Th1 cells, and their polyfunctional profile do not play a major role in determining severity of TB disease. This conclusion is in line with several recent clinical and experimental reports, suggesting that Th1 deficiency is not the initial factor of TB development (50, 51). The results also correspond to our previous analysis of IFN-γ levels measured in TBP in QFT assay: we found that antigen-driven IFN-γ secretion did not correlate with TB severity but mirrored disease activity increasing in a row: patients with residual TB lesions < patients with low disease activity < patients with high disease activity (52). Overall, patients with recent TB did not exhibit deficiency in *Mtb*-specific Th1 cells; the extent of Th1 response was associated with *Mtb* infection activity, but it did not correlate with TB disease severity.

In contrast to *Mtb*-specific populations of CD4^+^ lymphocytes that were not associated with TB severity, the whole lymphocyte population was a significant negative correlate of severe TB, especially, clinical TB severity (Tables 3 and 4). Mechanisms underlying this association are not fully clear. One possibility is that “unspecific” lymphocytes are not directly involved in the protection, and an association between their high percent/number and low TB severity is due to a bystander effect, i.e., to a concomitant decrease in “pathological” cell populations, such as neutrophils. However, in our study, lymphocyte and neutrophil populations associated with diverse TB manifestations. Another possibility to consider is a direct involvement of “non-specific” lymphocytes in host protection. Although the mechanisms for this effect are unclear, this possibility cannot be ruled out. Of note, it is in line with a recent hypothesis suggesting that acquired immunity against infectious diseases can in part be mediated by pathogen non-specific responses (53).

The next finding of the study is the demonstration of stable associations between pulmonary destruction and circulating neutrophils, particularly, band neutrophils (percent, numbers, and BLN). These results are in line with several other reports that demonstrated associations between neutrophilia and pulmonary TB severity (54–56). However, in most of these studies, TB severity was evaluated as an integrative/complex parameter raising a question on particular pathological processes that depended/were associated with neutrophilia. For example, in the study by Abakay et al., TB severity was evaluated based on the size, location, density, and destruction of TB lesions (55). In other studies, neutrophilia or high neutrophil:lymphocyte ratios were associated with TB mortality (54) or retreatment (56), both being integrative readouts of TB process. Recently, Lawn and co-authors reported high neutrophil counts to correlate with sputum *Mtb* positivity (57). This study, however, did not examine pathological processes ongoing in the lungs of enrolled patients. In our study, high neutrophil and, especially, band neutrophil counts correlated with both *Mtb* excretion and pulmonary destruction; the latter association was more statistically significant and likely primary (Table S2 in Supplementary Material).

Our finding on associations between neutrophils and pulmonary destruction raises questions on the role for neutrophils during TB, pathways mediating their pathological effects, and mechanisms leading to their accumulation.

The role for neutrophils in TB is debatable (58–60). Some studies demonstrated the involvement of neutrophils in granuloma formation, initiation of T-cell response, amplification of macrophage antimycobacterial activity, secretion of antimycobacterial peptides, and *Mtb* killing (61–64). In line with this, low levels of neutrophils and neutrophil-derived peptides have been associated with increased risk of TB (65, 66). Other studies, in contrast, demonstrated a strong association between neutrophilic inflammation and *Mtb* infection severity (67–71). To explain this inconsistency, we previously suggested that neutrophils may play different roles at different TB phases, mediating protection during the onset of the infection and inducing pathology at the advanced disease stage (72). However, in the current study, the correlation between neutrophils and TB severity was registered in patients with recent TB, indicating that neutrophil may contribute to TB pathology starting from the early stage of disease.

There are several mechanisms whereby neutrophils may contribute to TB pathology. Specifically, neutrophils were suggested to play a role of “Trojan horse” hiding *Mtb* from activated macrophages (59); together with their precursors, myeloid-derived suppressor cells, neutrophils may suppress T-cell responses (73); finally, neutrophils may directly mediate lung tissue damage. Our results on a strong correlation between the accumulation of neutrophils and the degree of lung tissue destruction support the latter mechanism. The involvement of neutrophils in pulmonary destruction during TB disease has been also suggested by Ong et al. who demonstrated elevated concentrations of neutrophil-derived metalloproteinase-8 in TBP with high radiological and clinical TB severities and the ability of metalloproteinase-8 to cause matrix destruction (74). Our results extend these findings by (i) dissecting pulmonary destruction from other radiological and clinical manifestations of TB severity and (ii) showing an additional association between pulmonary destruction and a particular neutrophil subset, i.e., band neutrophils. Yet, it should be emphasized that correlation does not mean causality and that correlation between band neutrophils and lung destruction could arise due to impact of TB on bone marrow function, and thus, band neutrophils could be indicators and not a cause of pulmonary destruction in TB.

The current study has some limitations. First, it is focused on circulating blood cells. We cannot exclude that lack of correlation between pools of *Mtb*-specific CD4^+^ cells and TB severity may result from the preferential accumulation of antigen-specific cells at the site of infection. However, in our study, TBP exhibited significant variability in the quantities and polyfunctional profile of circulating *Mtb*-specific CD4^+^ cells (see Figure 2). Thus, the study still demonstrates that this variability is not associated with TB manifestations. Second, in this study, we did not address fine mechanisms underlying an association between band neutrophils and pulmonary destruction and between bulk lymphocytes and low severity of several TB manifestations. These questions are in the focus of our ongoing studies.

Overall, our study provides new insight into several aspects of TB immunology. Specifically, it demonstrates that (i) clinical severity of TB is not associated with a degree of pulmonary destruction, *Mtb* excretion or TB extent; (ii) quantitative parameters of *Mtb*-specific CD4^+^ T cells and their polyfunctional profile differ between TBP and healthy participants, including LTBI subjects, but play a minor role in determining TB severity; (iii) pulmonary destruction, one of the leading causes of *Mtb* excretion, infection dissemination, and patient death, is tightly associated with an increase in band neutrophil population. Overall, the results indicate that severe TB develops as a result of pathological host reactivity to the infection rather than a deficiency in protective *Mtb*-specific Th1 responses and suggest a new factor of TB pathogenesis, i.e., alterations in granulocytic and lymphocytic lineages of differentiation. The study also points that detailed evaluation of TB process and comparison of immunological parameters in TBP exhibiting diverse TB manifestations is a valuable approach to address immune mechanisms and correlates of TB protection and pathology.

## Ethics Statement

The study was approved by the IRB (Institutional Review Board) no 1 of the Central Tuberculosis Research Institute (CTRI, Moscow), performed during years 2014–2016, and conducted in accordance to the principles expressed in the Declaration of Helsinki. All participants gave written informed consent to participate in the study.

## Author Contributions

AP acquired, analyzed, and interpreted all immunological data. IN analyzed and interpreted immunological data, performed data analysis in R, and contributed to the writing of the manuscript; TR, GK, and YS contributed to the acquisition and analysis of immunological data. IB and TB acquired and analyzed clinical data. RA and PS acquired and analyzed radiological data. EL acquired and analyzed microbiological data. LC analyzed microbiological data, contributed to the conception of the work and interpretation of microbiological data. VG analyzed and interpreted the data, performed data analysis in R, and wrote the manuscript. IL designed the study, contributed to data analysis, wrote the manuscript, and was the primary mentor on the project. AP and IN contributed equally to the work.

## Conflict of Interest Statement

The authors declare that the research was conducted in the absence of any commercial or financial relationships that could be construed as a potential conflict of interest.
