# Supplementary figures and images for: Severe Tuberculosis in Humans Correlates Best with Neutrophil Abundance and Lymphocyte Deficiency and Does Not Correlate with Antigen-Specific CD4 T-Cell Response
Source: Front Immunol. 2017 Aug 21;8:963. doi: 10.3389/fimmu.2017.00963 (PMC5566990; doi:10.3389/fimmu.2017.00963)

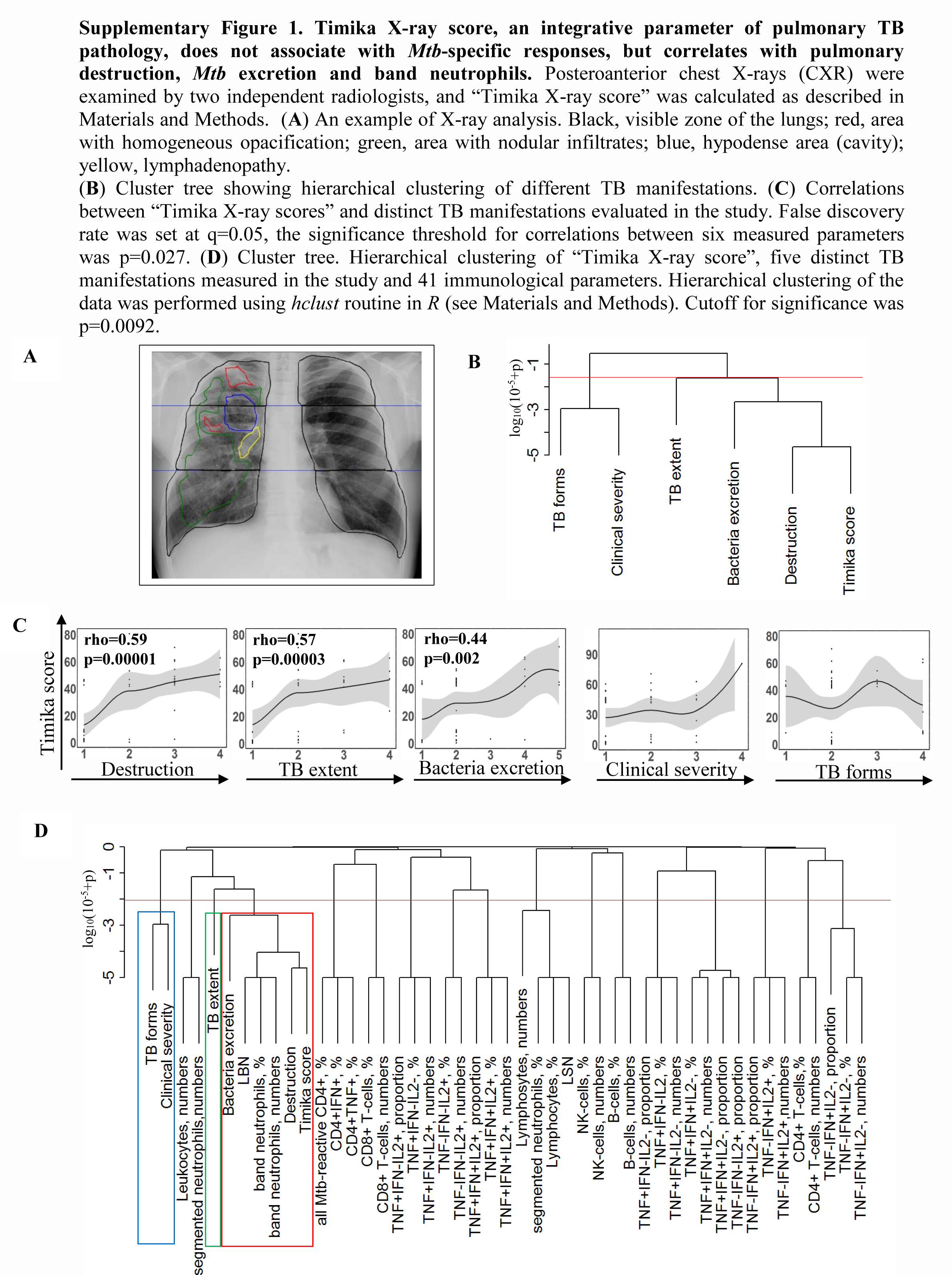

Supplement: Supplementary file 1 [file image_1.tif]
