# Supplementary material for: Severe Tuberculosis in Humans Correlates Best with Neutrophil Abundance and Lymphocyte Deficiency and Does Not Correlate with Antigen-Specific CD4 T-Cell Response
Source: Front Immunol. 2017 Aug 21;8:963. doi: 10.3389/fimmu.2017.00963 (PMC5566990; doi:10.3389/fimmu.2017.00963)
Supplement: Supplementary file 2 [file table_1.docx]

**Supplementary Table 1. Immunological parameters analyzed in the study**

Blood samples were obtained from TB patients and: a) stimulated with PPD in the presence of Brefeldin A, stained with mAb to CD4, IFN-γ, TNF-α and IL-2 and analyzed by flow cytometry; b) stained with mAbs to CD4, CD8, CD19 and CD16/CD56 and analyzed by flow cytometry; c) used for hematology test. The list of analysed parameters is presented. Frequency, cell percent out of all CD4^+^ lymphocytes; proportion, cell percent out of all *Mtb*-responding CD4^+^ cells.

| Group of parameters | Cells | Parameters analyzed* |
| --- | --- | --- |
| *Mtb*-responding  CD4^+^ cells | IFN-γ^+^ | Frequency, number |
|  | TNF-α^+^ | Frequency, number |
| Functional  subpopulations of *Mtb*-responding  CD4^+^ cells | TNF-α^+^IFN-γ^-^IL-2^-^ | Frequency, proportion, number |
|  | TNF-α^+^IFN-γ^-^IL-2^+^ | Frequency, proportion, number |
|  | TNF-α^+^IFN-γ^+^IL-2^-^ | Frequency, proportion, number |
|  | TNF-α^+^IFN-γ^+^IL-2^+^ | Frequency, proportion, number |
|  | TNF-α^-^IFN-γ^-^IL-2^+^ | Frequency, proportion, number |
|  | TNF-α^-^IFN-γ^+^IL-2^-^ | Frequency, proportion, number |
|  | TNF-α^-^IFN-γ^+^IL-2^+^ | Frequency, proportion, number |
| All *Mtb*-responding  CD4^+^ cells | The sum of seven functional subpopulations of *Mtb*-responding  CD4^+^ cells | Frequency, number |
| Lymphocyte subpopulations | CD4^+^ | Percent, number |
|  | CD8^+^ | Percent, number |
|  | CD19^+^ | Percent, number |
|  | CD16/CD56^+^ | Percent, number |
| Leukocyte populations | Leukocytes | Number |
|  | Segmented neutrophils | Percent, number |
|  | Band neutrophils | Percent, number |
|  | Lymphocytes | Percent, number |
