# Supplementary material for: Severe Tuberculosis in Humans Correlates Best with Neutrophil Abundance and Lymphocyte Deficiency and Does Not Correlate with Antigen-Specific CD4 T-Cell Response
Source: Front Immunol. 2017 Aug 21;8:963. doi: 10.3389/fimmu.2017.00963 (PMC5566990; doi:10.3389/fimmu.2017.00963)
Supplement: Supplementary file 3 [file table_2.docx]

**Supplementary Table 2. Minimal models explaining the variability in leukocyte populations in TB patients**

TB manifestations were evaluated and scored as shown in Table 2. Blood cells obtained from TB patients were analyzed for leukocyte populations, lymphocyte populations, *Mtb*-specific cytokine-producing cells. The following immunological parameters were included in the analysis as dependent variables: leukocyte counts, segmented neutrophils percent and numbers, band neutrophil percent and numbers, lymphocyte percent and numbers; the frequency of CD4+, CD8+, CD19+ and CD16/CD56+ cells; the frequency of all *Mtb*-specific cells, TNF-α+, IFN-γ+ lymphocytes; the frequencies of seven polyfunctional subpopulations of *Mtb*-specific cells. Before performing regression analysis, data were normalized, and for each immunological parameter full and minimal models were generated. Full models included the following variables: destruction, TB extent, clinical severity, bacteria excretion, TB forms, and the following interactions: TB forms/clinical severity, destruction/ bacteria excretion, TB extent/bacteria excretion, TB forms/TB extent. Minimal models that did not differ from full models and minimal models with the adjusted R-squared <0.10 are not shown.

| **Predictors** | **Estimate** | **Std. Error** | **t-value** | **Pr(>\|t\|)** | **Adjusted R-squared** |
| --- | --- | --- | --- | --- | --- |
| **Leukocyte counts** | | | | | |
| (Intercept) | 24.633 | 6.612 | 3.726 | 0.000522 |  |
| Destruction | 5.075 | 2.933 | 1.730 | 0.090188 |  |
| Bacteria excretion | 5.478 | 2.741 | 1.999 | 0.051454 | **0.2013** |
| **Segmented neutrophils, percent** | | | | | |
| (Intercept) | -0.2121 | 18.5632 | -0.011 | 0.9909 |  |
| TB form | 7.1925 | 8.2474 | 0.872 | 0.3878 |  |
| TB extent | 9.0668 | 2.9516 | 3.072 | **0.0036** |  |
| Clinical severity | 28.6514 | 9.9913 | 2.868 | **0.0063** |  |
| TB form:  Clinical TB severity | -8.3754 | 4.0553 | -2.065 | **0.0447** | **0.2524** |
| **Segmented neutrophils, numbers** | | | | | |
| (Intercept) | 21.839 | 8.722 | 2.510 | 0.0158 |  |
| TB form | -5.630 | 3.548 | -1.587 | 0.1197 |  |
| TB extent | 4.475 | 3.068 | 1.459 | 0.1518 |  |
| Destruction | 4.970 | 2.875 | 1.729 | **0.0908** |  |
| Bacteria excretion | 4.886 | 2.652 | 1.842 | **0.0722** |  |
| Clinical severity | 5.066 | 3.529 | 1.436 | 0.1582 | **0.2880** |
| **Band neutrophils, percent** | | | | | |
| (Intercept) | 25.987 | 5.205 | 4.993 | 8.25e-06 |  |
| Destruction | 10.539 | 2.261 | 4.662 | **2.53e-05** | **0.2973** |
| **Band neutrophils, numbers** | | | | | |
| (Intercept) | 19.384 | 5.974 | 3.245 | 0.00217 |  |
| Destruction | 9.396 | 2.650 | 3.545 | **0.00090** |  |
| Bacteria excretion | 3.885 | 2.476 | 1.569 | 0.12342 | **0.3487** |
| **Lymphocytes, %** | | | | | |
| (Intercept) | 109.985 | 16.606 | 6.623 | 4.09e-08 |  |
| TB form | -9.422 | 7.421 | -1.270 | 0.2108 |  |
| Destruction | -5.630 | 2.353 | -2.393 | **0.0211** |  |
| TB extent | -9.455 | 2.781 | -3.400 | **0.0014** |  |
| Clinical severity | -24.991 | 9.095 | -2.748 | **0.0087** |  |
| TB form:  Clinical severity | 7.605 | 3.660 | 2.078 | **0.0436** | **0.4201** |
| **Lymphocytes, numbers** | | | | | |
| (Intercept) | 101.298 | 19.625 | 5.162 | 5.36e-06 |  |
| TB form | -13.461 | 8.719 | -1.544 | 0.1296 |  |
| TB extent | -6.269 | 3.120 | -2.009 | **0.0506** |  |
| Clinical severity | -22.517 | 10.563 | -2.132 | **0.0385** |  |
| TB form:  Clinical severity | 6.602 | 4.287 | 1.540 | **0.1306** | 0.1884 |
| **CD4^+^, percent** | | | | | |
| (Intercept) | 61.173 | 8.775 | 6.971 | 1.01e-08 |  |
| TB form | -5.757 | 3.914 | -1.471 | 0.1481 |  |
| TB extent | 5.774 | 3.213 | 1.797 | 0.0789 |  |
| Clinical severity | -7.236 | 3.797 | -1.906 | 0.0629 | 0.1214 |
| **All *Mtb*-reactive** | | | | | |
| (Intercept) | 26.759 | 17.906 | 1.494 | 0.1422 |  |
| TB form | -8.775 | 3.598 | -2.439 | 0.0188 |  |
| Destruction | 9.953 | 7.115 | 1.399 | 0.1689 |  |
| Bacteria excretion | 13.681 | 8.183 | 1.672 | 0.1016 |  |
| Clinical severity | 10.493 | 3.865 | 2.715 | **0.0094** |  |
| Destruction:  Bacteria excretion | -5.278 | 2.920 | -1.808 | 0.0775 | 0.1468 |
| **TNF-α^+^, frequency** | | | | | |
| (Intercept) | 23.603 | 18.106 | 1.304 | 0.1992 |  |
| TB form | -7.825 | 3.638 | -2.151 | 0.0370 |  |
| Destruction | 9.096 | 7.194 | 1.264 | 0.2128 |  |
| Bacteria excretion | 11.907 | 8.274 | 1.439 | 0.1572 |  |
| Clinical severity | 10.716 | 3.908 | 2.742 | **0.0088** |  |
| Destruction:  Bacteria excretion | -4.065 | 2.952 | -1.377 | 0.1755 | 0.1275 |
| **TNF-α^+^IFN-γ^-^IL-2^-^, frequency** | | | | | |
| (Intercept) | 49.710 | 25.129 | 1.978 | 0.0543 |  |
| TB form | -19.410 | 10.707 | -1.813 | 0.0768 |  |
| TB extent | -5.225 | 11.104 | -0.471 | 0.6403 |  |
| Bacteria excretion | 13.786 | 8.641 | 1.595 | 0.1179 |  |
| Clinical severity | 12.909 | 4.003 | 3.225 | **0.0024** |  |
| TB form:  TB extent | 6.261 | 3.947 | 1.586 | 0.1200 |  |
| TB extent:  Bacteria excretion | -6.275 | 3.852 | -1.629 | 0.1106 | 0.1363 |
| **TNF-α^+^IFN-γ^+^IL-2^-^, frequency** | | | | | |
| (Intercept) | 24.1724 | 27.0087 | 0.895 | 0.3759 |  |
| TB form | -12.5250 | 11.8079 | -1.061 | 0.2949 |  |
| TB extent | 0.4059 | 11.2138 | 0.036 | 0.9173 |  |
| Bacteria excretion | 16.1533 | 8.4979 | 1.901 | 0.0642 |  |
| Clinical severity | 27.2366 | 11.5728 | 2.354 | **0.0234** |  |
| TB form:  TB extent | 5.9582 | 4.0556 | 1.469 | 0.1493 |  |
| TB form:  Clinical severity | -6.552 | 4.6647 | -1.405 | 0.1673 |  |
| TB extent:  Bacteria excretion | -6.7634 | 3.7775 | -1.790 | 0.0806 | 0.1714 |
| **TNF- α ^–^IFN-γ^+^IL-2^+^, frequency** | | | | | |
| (Intercept) | 17.515 | 19.754 | 0.887 | 0.3800 |  |
| TB form | 19.769 | 9.078 | 2.178 | 0.03471 |  |
| Bacteria excretion | -7.320 | 2.563 | -2.856 | 0.00647 |  |
| Clinical severity | 22.840 | 11.119 | 2.054 | 0.04580 | 0.1339 |
